# Assessment and Management of Cognitive and Psychosocial Difficulties for People with Multiple Sclerosis in Ireland: A National Survey of Clinical Practice

**DOI:** 10.1155/2022/3232076

**Published:** 2022-10-21

**Authors:** Sinéad M. Hynes, Fiadhnait O'Keeffe, Eimear Bane, Megan H. Oglesby, Christopher P. Dwyer, Robert Joyce, Olga A. Klein

**Affiliations:** ^1^Discipline of Occupational Therapy, School of Health Sciences, National University of Ireland Galway, Galway, Ireland; ^2^St. Vincent's University Hospital, Dublin, Ireland; ^3^School of Social Science, Technological University of the Shannon IE, Athlone, Ireland; ^4^German Center for Neurodegenerative Diseases, Rostock, DE, Germany

## Abstract

**Background:**

A recent survey of 109 healthcare professionals explored how UK healthcare professionals typically assess and treat multiple sclerosis (MS)-related cognitive impairment. Little is currently known about what constitutes usual care for cognitive impairment and psychosocial care for people with MS in Ireland.

**Aim:**

The aim of the current research was to survey healthcare professionals (HCPs) who work with people with MS, to understand current assessment and management of cognition and psychosocial care in people with MS in the Republic of Ireland.

**Methods:**

A cross-sectional survey design was used. Data were collected online through Microsoft forms and through postal responses. The original UK questionnaire was adapted, piloted, and distributed to Irish HCPs. Participants were qualified HCPs who work clinically with people with MS in the Republic of Ireland.

**Results:**

Ninety-eight HCPs completed the survey. Only 34% of those surveyed reported routine screening of cognition for people with MS within their services; approximately, 36% HCPs reported that they did not provide information or services in relation to cognition to people with MS and 39% reported not referring elsewhere when cognitive difficulties were suspected. Out of the 98 HCPs, 47% reported assessing mood difficulties as part of their services, with 14% unsure. In total, 70% of participants reported onward referral took place if mood difficulties were identified. The Montreal Cognitive Assessment was the most commonly administrated cognitive assessment. Cognitive intervention choices were found to be guided by clinical judgement in 75.5% of cases. *Discussion*. Despite the high importance placed on cognitive and psychosocial care, there is very little consistency in treatment and assessment across services for people with MS in Ireland.

## 1. Introduction

Cognitive impairment affects 40 to 65% of people with multiple sclerosis (MS) [1, 2] and can impact memory, attention, processing speed, and executive functioning [3]. These deficits can in turn have a significant impact on individuals' quality of life [4] and ability to remain in employment [5]. Specifically, only 43% of people of working age with MS are in employment in Ireland, compared with the 60% average of people with MS in the EU [6]. Moreover, a recent Irish study found participants with MS were dissatisfied with the lack of intervention and general awareness regarding cognitive difficulties in MS [7]. Currently, little is known about what constitutes ‘usual care' of cognitive impairment in people with MS in Ireland. As such, a greater understanding of the assessment and treatment of cognitive impairments is overdue in an Irish context.

With respect to assessment, a number of evidence-based cognitive test batteries for people with MS are recommended for clinical practice, including the Minimal Assessment of Cognitive Function in MS MACFIMS [8], the Brief International Cognitive Assessment for MS BICAMS [9], and the Brief Repeatable Battery of Neuropsychological Tests BRB‐N; [10]—all of which assess cognitive impairments most commonly observed in people with MS [11]. These assessments have all been found to be sensitive, specific, and reasonably short [12]. The BICAMS has also been validated in an Irish population [13]. The Symbol Digit Modality Test (SDMT), a single measure of processing speed that is included as a test in the BICAMS, BRB-N, and the MACFIMS, has also been recommended as a quick and valid screening tool for detecting cognitive impairment in people with MS [3, 14]. It is, however, unclear how or to what extent and which cognitive assessments are currently being used in Ireland by HCPs working with people with MS.

A recent survey [15] explored how UK healthcare professionals (HCPs) typically assess and treat MS-related cognitive impairment. They found great variety in the assessment of cognitive symptoms in people with MS across the UK, with the Montreal Cognitive Assessment (MoCA) the most commonly used by clinicians (i.e., 41.3%). According to Klein and colleagues [15], of the 109 responses from HCPs, 50 different assessments were reported as being used. Of the assessment tools that are widely recommended for people with MS, only one was reported as being used regularly (i.e., the BRB-N, used by 11% of respondents) and the single measure tool, the SDMT, was reported as being used by 5.5% of respondents. The BICAMS and the MACFIMS, assessments that were designed and validated for people with MS, were reported as being among the least used assessment tools by HCPs, suggesting that evidence-based assessments may not be widely used in clinical practice.

Klein et al. [15] also examined what intervention or treatment, if any, was offered to people with MS in the UK. HCPs reported advising patients to use external memory strategies such as alarms and diaries, with minority educating patients on internal memory strategies, like mnemonics, repetition, and rehearsal. Notably, while external memory aids are useful tools to help improve everyday functioning, they do not improve memory [16], whereas internal memory strategies such as rehearsal and mnemonics have been found to improve memory in those with cognitive impairments [17, 18] and those with MS with cognitive impairment [19–21]. HCPs in the UK reported a lack of confidence in managing cognitive impairment, which was associated with a lack of post-registration training in cognitive rehabilitation [15].

There has been an emerging evidence base into the effects of cognitive rehabilitation in people with MS, with some benefits found in programs that focused on specific cognitive domains, such as executive function, processing speed, and attention [22, 23]. A recently published Cochrane review of memory rehabilitation for people with multiple sclerosis [24], which included 44 studies of 2714 participants, found promising short-term results (1–6 months postintervention) in subjective memory, quality of life, verbal memory, and information processing, with recommendations for further research needed. A meta-synthesis of the experiences of people with MS who participate in cognitive rehabilitation programs found benefits of the group environment, emotional and social improvements, as well as increased knowledge, and understanding of cognition [25]. Improved confidence and quality of life following cognitive and memory rehabilitation could have substantial impacts on daily functioning for people with multiple sclerosis who experience cognitive difficulties. Though MS Ireland, the national organization in Ireland that provides support and information to people living with MS, recommends people with cognitive issues attend neuropsychological rehabilitation, it is unclear what form of cognitive rehabilitation is being used by HCPs in Ireland and how it compares to extant evidence.

People with MS are up to three times more likely to experience depression than those in the general population, with estimates of between 30% and 50% prevalence rates [26, 27]. Anxiety is also commonly reported in people with MS, with prevalence rates reported between 22% and 41% [26, 28]. For people with MS, experiencing depression and anxiety is associated with reduced quality of life, disability progression, increased pain, and increased health care utilization [28–30]. Mood disorders tend to be underidentified and undertreated in MS leading to low access to effective psychological and psychopharmacological treatments [31].

Screening for mood disorders is recommended for all those with chronic health conditions [32]. Annual screening for mood difficulties is recommended for people with MS to identify any changes in mood that in turn may be having a negative impact on cognition [11]. It is recommended that discussions regarding mood and mental health be incorporated into routine MS care. Clinical interview and regular use of screening tools are advised to monitor the presence of and any changes in mental health in people with MS. Onward referral to specialists is advised when necessary [33].

There is also a complex interplay between mood, fatigue, and cognition in MS. Depression and anxiety are typically associated with poorer memory, processing speed, and executive functioning in people with MS [11, 34, 35]. Depression has also been strongly associated with subjective or self-reported cognitive functioning in people with MS [36–38].

As it is not currently known what approach and methods of MS-related assessment and management of cognitive and mood symptoms in MS are used in Ireland, the current study aims to replicate and adapt the UK survey [15] in order to better understand these in an Irish context. The study will also explore MS HCPs' perception of the importance of these issues and their confidence in assessing and managing cognitive difficulties. The survey is also expanded to include questions regarding the assessment and management of psychosocial issues related to MS, given the impact on cognition and quality of life. Such knowledge is vital to maximize current interventions available to people living with MS, as well its impact on current and future research in the field.

Thus, the objectives of the current research were to develop an understanding of the following conditions:

1. Current assessment methodologies of cognitive impairment and psychosocial difficulties in people with MS in the Republic of Ireland
2. Current clinical practice in the management of cognitive impairment and psychosocial difficulties in people with MS

## 2. Methodology

A cross-sectional survey design was used to achieve the research aims. Ethical approval for this research was obtained.

### 2.1. Data Collection Tool

The original questionnaire developed and administered by Klein et al. [15] was adapted, with permission, as appropriate to the Irish context and expanded to include specific questions regarding the assessment and intervention of psychosocial difficulties.

The adaptation process was completed with input from the original author (O.A.K.) as well as HCPs, and researchers, and with the patient and public involvement. The questionnaire addresses general information regarding symptoms assessed and treated within the Irish Health Service Executive (HSE); assessment practices of cognitive difficulties and mood in people with MS; management practices of cognitive difficulties and mood in MS; usual care for cognition and mood; and HCP perception of this routine. The questionnaire also addresses the HCPs' professional background, training, and role within a service; clinical pathways and referral methods; the HCPs' confidence in assessing and managing cognitive difficulties in people with MS; and HCPs' perceptions of the importance of assessing and managing cognitive difficulties in people with MS.

Prior to administration for data collection, the questionnaire was initially piloted with a small sample of people (who met all the inclusion criteria apart from working with people with MS) who were not included in the main study, in order to examine the viability, feasibility, and accessibility of the questionnaire [39]. Following piloting, minor changes were made to the order and phrasing of some questions.

### 2.2. Participants

Participants were qualified HCPs who work clinically with people with MS in the Republic of Ireland, including (but not limited to) occupational therapists, neuropsychologists, clinical psychologists, physiotherapists, MS nurses, and neurologists. Potential participants were excluded if they had no experience of working clinically with people with MS.

### 2.3. Procedure

HCPs were recruited through the respective professional bodies of HCPs, MS Ireland, and Special Interest Groups. HCPs were advised that they could inform other potentially interested colleagues of the study, to allow snowball sampling. Social media (in particular Twitter) were also used to recruit relevant HCPs. The online questionnaire was hosted through Microsoft forms.

Notably, during the course of data collection, the HSE was the target of a cyber-attack. As a result, many HCPs had no access to computers and/or the Internet for at least three months (May-July, 2021). As a result, HCPs were offered the option of completing a paper copy of the questionnaire, with copies posted to hospital and rehabilitation units (*n* = 86) including respite units, and primary care centers (*n* = 127) in the Republic of Ireland. Letters were sent to a named HCP in that organization and included two copies of the questionnaire, allowing for photocopying of the questionnaire, if needed.

### 2.4. Analysis

The responses from Microsoft forms were exported into an Excel file and screened for errors and omissions to ensure data integrity. Data yielded from the questionnaires were analyzed both quantitatively and qualitatively. Descriptive statistics were calculated and data gathered from the open-ended questions was analyzed using an adapted version of the content analysis process outlined by Bengtsson [40]. This was completed through the following steps: 1) Becoming familiar with the data. 2) Meaning units were identified from the text, compared with the original text, and data was then organized under headings and subheadings. 3) Ensure all relevant data had been extracted. 4) Findings were reported in parallel with quantitative data to provide further detail, depth, and context, where appropriate.

## 3. Results

Ninety-eight participants (79 completed online; 19 postal) completed the survey between February and May, 2021^1^. Of the 95 participants who provided their professional background, 34 were occupational therapists, 20 were physiotherapists, 13 were nurses (6 MS specialist nurses, 5 general nurses, 2 neurology nurse specialists), 11 psychologists (5 clinical neuropsychologists, 3 clinical psychologists, and 3 assistant psychologists), 6 were neurologists, and 4 were speech and language therapists. The other professions that made up the participants were clinical research fellow (*n* = 2), social worker (*n* = 2), audiologist (*n* = 2), and MS case-worker (*n* = 1). The number of years healthcare professionals had been working with people with MS is shown in Table 1.

**Figure 1:**
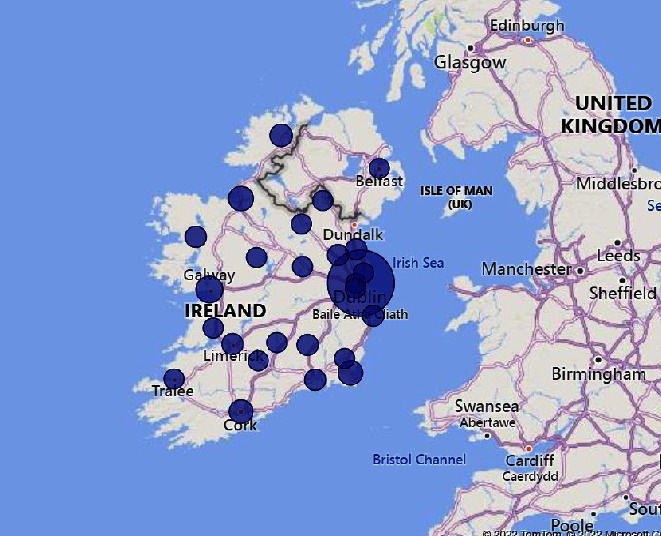
A heat map of where participants were working across the country. A heat map of survey participants.

The healthcare professionals surveyed worked in various settings across the country. Out of 71 participants, 24 worked in a primary care, 15 worked in inpatient neurological rehabilitation, 14 were based in MS clinics, 7 worked in outpatient neurological rehabilitation, and 7 worked in community rehabilitation teams (neuro and nonspecific), long stay, respite (*n* = 6), and acute hospital (*n* = 5). Other settings listed included adult disability team (*n* = 2), assessment services for assistive technology and seating (*n* = 1), casework (*n* = 1), older person's inpatient rehabilitation service (*n* = 1), physiotherapy service (*n* = 1), and continence advisory service (*n* = 1) (See Figure 1).

With respect to post-registration training (see Table 2), the format of the training varied across those who had received training. Seventeen of the healthcare professionals named in-house training or short courses, 12 people had gained a relevant academic qualification, while another 12 had attended a day courses or conference. Four participants had completed the online training, three had learned from colleagues, and two people reported self-directed learning.

### 3.1. Professional Makeup of the Multidisciplinary Team

Participants were asked what HCPs made up their multidisciplinary team (MDT) that worked with people with MS. Physiotherapists were the most commonly reported HCP named on MDTs (*n* = 85), with occupational therapists the second most commonly reported HCP (*n* = 71). The other MDT professions named were nurses (*n* = 61), with MS specialist nurses listed separately by 35 participants; speech and language therapists (*n* = 54); neurologists (*n* = 40); clinical neuropsychologists (*n* = 21); clinical psychologists (18); assistant psychologists (*n* = 13); (medical) social worker (*n* = 8); dietician (*n* = 3), healthcare assistant (*n* = 3); rehabilitation consultant (*n* = 3); and case manager (*n* = 2). One participant stated that they worked alone. The services offered by the MDTs to address MS symptoms are listed in Table 3. No structured rehabilitation was reported by two HCPs, with a further two HCPs reporting onward referral here.

#### 3.1.1. Cognitive Care Pathways

**Figure 2:**
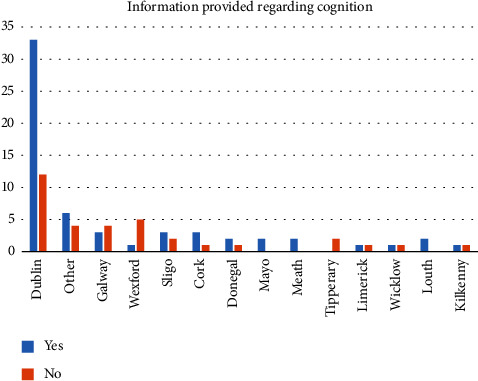
Services providing information regarding cognition according to HCPs surveyed.

Sixty-four percent (*n* = 60 of 94 respondents to question) of services reported providing information to patients about cognitive difficulties associated with MS. Thirty-six percent did not provide information.  Figure 2 charts this information provision across the counties that HCPs surveyed worked in (no response from four participants).

When asked what information is provided, thirty-seven participants stated that they provide written resources such as leaflets. Twenty-nine participants discuss and provide psychoeducation on an individual basis, and eighteen use online resources. Information about other cognitive services was provided to patients by 13 participants, while 11 refer directly on to other services. Eight participants reported providing in-service treatment or assessment, including strategies to manage cognitive difficulties. The following were specified by one participant each: education of family/friend; group-based discussions; counseling/emotional support; normalizing cognitive problems.

When asked if people with MS are routinely screened or assessed for cognitive difficulties, 57% of participants reported that they were not, 34% reported that they were, and 9% were unsure. When asked when in the service pathway routine screening normally happens, twenty-three participants said that people are screened on admission or during an initial assessment, while ten participants are assessed following the initial screening, if required. Thirteen participants stated that cognitive screening/assessment was conducted on a case-by-case basis, with eleven assessing if cognitive deficits become apparent. If cognitive issues are flagged by a patient of a family member (*n* = 6) or the place of referral (*n* = 3) that leads to the cognitive assessment taking place in some services. Five participants reported that cognitive screening rarely or never happens.

When asked how cognition was assessed in the service, 76 participants take a history from the patient and/or caregiver/family member, 56 participants use medical notes or referral letter, and 70 participants used screening measures/assessments. Other methods of assessing cognition listed were functional assessment (*n* = 4), informal assessment during activities of daily living (*n* = 2), not sure (*n* = 2), not assessed or “r*eally rare and really rare that we have the time to do this*” (*n* = 2; Physiotherapist ID20; Neurologist ID03). Twelve participants reported not assessing cognition in their service.

Referral for specialist cognitive assessment when cognitive difficulties were suspected was reported by 60% of participants (*n* = 37 within the service, and *n* = 20 in another service). Forty percent of participants do not refer for specialist assessment. The specialist or service listed by participants were psychology (*n* = 27; neuropsychologists *n* = 21; clinical psychologist *n* = 6), occupational therapists (*n* = 19), GP/neurologist (*n* = 14), memory clinic (*n* = 5), psychiatrist (*n* = 1), and nurse (*n* = 1).

#### 3.1.2. Cognitive Assessment

**Figure 3:**
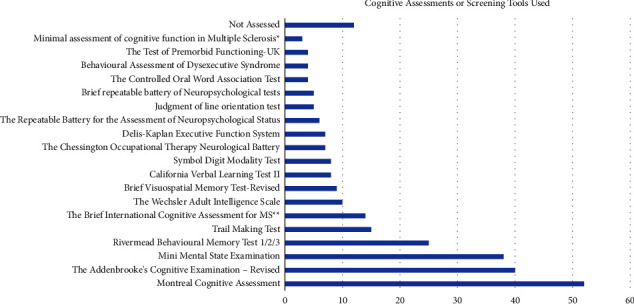
Assessments used by healthcare professionals to assess cognition in people with MS. ^*∗*^The MACFIMS consists of SDMT, PASAT, CVLT2, BVMTR, COWAT, JLO, and DKEFS sorting but is listed as a battery of assessments here. ^*∗∗*^The BICAMS consists of SDMT, CVLT2, and BVMTR but is listed as a battery of assessments here.

Thirty-nine assessments were identified by participants to assess cognition in clinical practice.  Figure 3 charts the most commonly used assessments (those named by a minimum of three HCPs), and Appendix 1 detailed those listed by fewer than three participants. The most commonly used cognitive assessment reported was the MOCA (*n* = 52), with the Addenbrookes Cognitive Examination-Revised (ACE-R; *n* = 40), the Mini-Mental State Examination (MMSE; *n* = 38), and the Rivermead Behavioral Memory Test (RMBT; *n* = 25) commonly used in clinical practice.

The identified HCPs in the MDT who primarily complete assessment of cognition are presented in Table 4. Occupational therapists are reported to conduct much of the cognitive assessment across the services surveyed (*n* = 56). If cognition is indicated to be an issue following assessment, specific next steps are taken by participants. The findings of the assessment are discussed with the individual (*n* = 75), the family and/or carer (*n* = 56), and the individual's GP is informed (*n* = 45), as well as other therapists involved with the individual (*n* = 63). There was an emphasis on team decision making in the “other” approaches that were mentioned by participants–for example, one participant said that.

*“Cognitive profile shared with team and strategies shared across disciplines”* (Physiotherapist, ID97).

Two participants mentioned that they had to make decisions themselves as they did not have a team, with one participant stating that they had no one to refer to for further input:

“*We are limited in what we can do as we have no one to refer to*” (Neurologist, ID03).

A number of the HCPs specifically address the cognitive difficulties through offering cognitive strategies secondary to other rehabilitation (*n* = 52), adapting how rehabilitation is delivered to the individual (*n* = 42), or developing and implementing a cognitive rehabilitation plan themselves (*n* = 23), or in conjunction with colleagues (*n* = 6).

Referral for specialist cognitive rehabilitation (*n* = 18) is completed by some, though thirty-two participants stated that no specialist rehabilitation is available to their patients. This point was made by one HCP participant who said

“*Many regions have no* neuropsychology *services for [people with MS]. Zero, nil. Mostly available only in the large urban centers*” (MS Case-worker, ID27).

Those who do refer do so to clinical neuropsychology (*n* = 15), occupational therapy (*n* = 8), neurology (*n* = 4), and GP (*n* = 3).

### 3.2. Cognitive Intervention

Participants were presented with a list of strategies and asked what, if any, they use with people with MS.  Table 5 presents the strategies and approaches identified, including internal strategies (those involving mental practice and rehearsal such as mnemonics), external strategies (compensatory approaches such as external memory aids), and teaching methods. For some of the strategies mentioned, participants elaborated on their use in clinical practice. One participant reported that some patients were reluctant to use technology to help with cognitive difficulties, as they did not want to become reliant on the technology.

“*I then mention how using* tools *can help, e.g., phone can help them achieve what they need to. Using tools does not make cognition weak, it can strengthen it by* maintaining *engagement and participation*” (Occupational therapist, ID26).

The importance of psychoeducation was emphasized, especially when healthcare professionals had limited contact with patients. One participant who reported limited contact with patients because of the demands of the services ensures that patients have the information they need on cognition following assessment. They have a number of key points of information that they tell the patients before providing any strategies, such as this occupational therapist.

*“1) There is more to cognition than memory alone. (2) MS can impact cognition and health professionals often do not acknowledge this enough, but it is important. (3) If you experience difficulties with your cognition the most common feeling around this is panic-do not panic, there are things that are effective that you can do to help”* (Occupational therapist, ID26).

When asked what guides the choice of strategy and approaches used, 74 HCPs stated that they use their clinical judgement, 18 follow a plan devised by another professional, 14 base their treatment on specific training they have received, and six HCPs follow a service-specific protocol. Two participants reported reading evidence on the topic, including NICE guidelines or a manualized rehabilitation program, and making decisions based on this. Another participant reported more of an ad hoc approach.

*“To be honest, I give advice but it is not grounded in a theory or programme, it's fairly ad hoc and I need to improve my education around this”* (MS Specialist Nurse, ID47).

**Figure 4:**
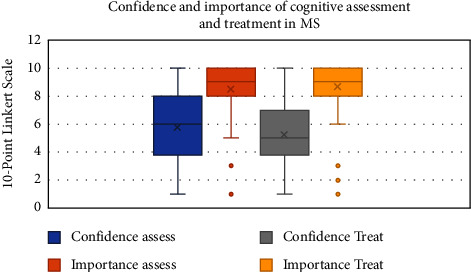
Healthcare professional's rating of importance and confidence in cognitive care.

Similar to this concept, HCPs were also asked to rate both their confidence and the importance they attach to the assessment and treatment of cognitive difficulties in MS (see Figure 4). Mean confidence levels to assess and treat cognitive difficulties were 5.787 (SD 2.66) and 5.245 (SD 2.43), respectively. The mean importance of assessment and treatment of cognitive difficulties were 8.479 (SD 1.92) and 8.67 (SD 1.86), respectively.

Eight of the HCPs stated that they would like access to further education, for example, “*training to OT staff in Ireland is limited*” (occupational therapist, ID62). Participants expressed a desire to link with other professionals working in the area, as well as having access to courses and training specific to the needs of people living with MS, especially in the assessment and intervention in the areas of mood and cognition.

*“Courses and training on working with people with MS and cognitive difficulties would be welcomed for general services such as primary care”* (Occupational therapist, ID34).

#### 3.2.1. Psychosocial Care

Results found that 47% (*n* = 45) of participants reported screening patients with MS for mood difficulties, 39% (*n* = 37) of HCPs said they did not screen for mood difficulties, and 14% (*n* = 13) of participants were unsure. When asked at what point in the service pathway people are screened for mood difficulties, participants reported the following: on admission or first appointment (*n* = 25), at clinical review (*n* = 18), informally reviewed at unspecified times (*n* = 8), if referred for assessment (*n* = 6), on an ongoing basis (*n* = 6), and only if necessary or if flagged (*n* = 5).

When asked what HCP usually screens for mood difficulties, a variety were listed (see Table 6).

**Figure 5:**
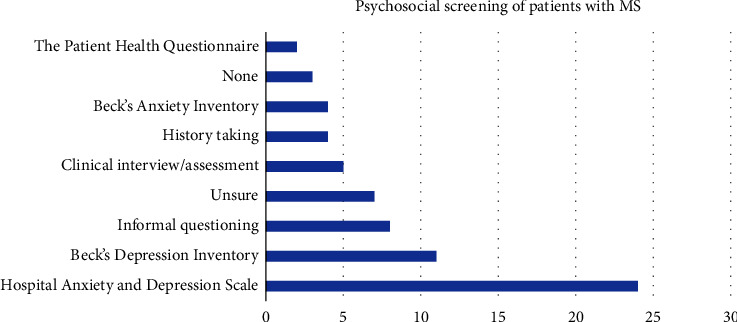
Mood screening tools/approaches listed by healthcare professionals.

The screening tools used to assess psychosocial functioning were listed by 52 participants (see Figure 5 for the most commonly reported and Appendix 1 for assessments listed once).

**Figure 6:**
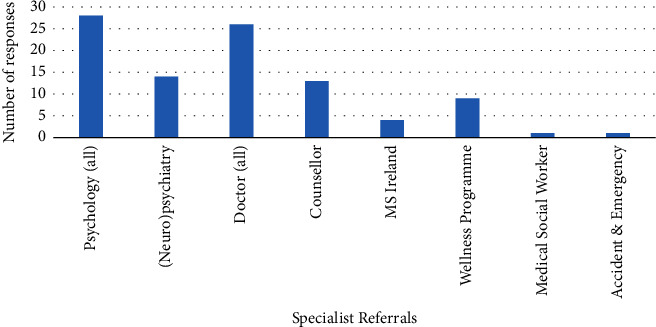
Specialists referred to by participants when mood difficulties were identified in people with MS.

If mood difficulties were identified during screening, 70% (*n* = 60) of HCPs refer on for further consultation, while 8% (*n* = 7) do not, and 22% (*n* = 19) reported being unsure.  Figure 6 presents specialist services referred to when psychological issues were identified.

People with MS were routinely provided information about psychosocial and mood difficulties according to 22% (*n* = 20) of HCPs. No information is routinely provided according to 49% (*n* = 46) of HCPs and 29% (*n* = 27) of participants were unsure. When asked what specific intervention pathways some ad hoc approaches were described by participants. Some reported liaising with medical social workers or psychologists one-to-one and referring participants to group education and self-management programs run by the HSE.

*“Depends on issues > if neuropsychology deems appropriate, offered intervention through them or through local counselling services, online information”* (Neurologist, ID08).

Some participants provided more specific details:

*“The intervention depends on the level of mood difficulty. If it can be managed by the GP or community services then we refer back to them. If it seemed more significant we refer to the neuro psychiatrist”* (MS Specialist Nurse, ID02).

#### 3.2.2. HCP Views on Existing Services for People with MS

At the end of the survey, participants were asked if they had anything further they would like to add. Nine participants spoke about the lack of essential services that are available for people with MS. Participants stated that there were limited MS specialist roles across all disciplines. The impact of this on services was discussed. One example was the lack of social workers in this area:

*“Insufficient social work resources to meet a patient psychosocial need in acute or community services. Increase in this resource would advocate for patient rights, psychosocial involvement including championing rights for appropriate services for persons experiencing cognitive decline, low-level mood or mental health decline and health promotion and social integration”* (Social worker, ID09).

Long waiting lists and limited access to services were areas highlighted by five participants. The HCPs stated that even if they were to assess for psychosocial or cognitive problems, they cannot provide the interventions needed because of long waiting lists. The same issue was mentioned when the participants refer onward for intervention.

*“If we screen for cognition changes then referral for neuropsychological assessment is a long wait for the patient”* (Neurology Nurse Specialist, 078).

Another five people indicated that there was a need for a specific pathway from assessment to rehabilitation for people with MS.

*“It would be great to have a pathway for a standardised assessment, intervention programme and outcome measures specific to this client group”.* (Occupational therapist, ID60).

The need for specific rehabilitation service was also emphasized.

*“We do not really have a rehabilitation service at all for people with MS in any sort of a structured way.”* (Neurologist, ID03).

Finally, the issue of geographical disparity was discussed by three participants. The potential of having a remote service to address the “*longstanding issue in regional Ireland*” (Occupational therapist, ID26) was discussed because of the limited rehabilitation services available outside of the main cities.

*“There is a disparity and inequity for people with MS to clinical support services across geographical areas.”* (Occupational therapist, ID33).

## 4. Discussion

This survey provides an overview of the current assessment and management of cognitive and mood difficulties for people with MS living in Ireland. There was a geographical spread in the respondents, though the majority were based in Dublin. Occupational therapists and physiotherapists were the highest represented HCPs surveyed, and from a service setting perspective, many respondents worked in primary care or inpatient rehabilitation centers. Occupational therapists were also the profession most likely to assess and treat cognitive difficulties, which was also reported by Klein and colleagues [15]. A number of healthcare professionals did not anticipate would be involved in the cognitive care of people with MS were captured in the “Other” section of the survey. Some of these professionals were assistants to other HCPs and so could be involved in this area under the guidance of another HCP, such as an occupational or physiotherapist. Other HCPs such as pharmacists might be interested in participating because of the potential impact of some disease-modifying therapies on cognition, e.g., anticholinergic agents.

A total of 39 separate cognitive assessments were named by HCPs in the survey. Similar to the UK study of cognitive management [15], the MOCA, ACE-R, and the MMSE were the most commonly reported cognitive assessments used with people with MS. Internationally, the assessment batteries that are recommended for use in cognitive care of people with MS are the MACFIMS [41], and the BICAMS [9], which were reported to be used by only 17 participants in the current study. In separate research from Finland and the US, there appears to be a trend toward increased neuropsychological testing for people with multiple sclerosis [42, 43]. Given that there is no existing research from Ireland on this topic, it is not clear if the results reported here reflect a change toward increased cognitive testing.

There appears to be a focus on cognitive screening that is quick and easily administered by HCPs surveyed. These assessments, though commonly used in practice, were not developed for use with people with MS. The MOCA, for instance, is recommended for use with people with mild cognitive impairment [44] and does not assess the speed of information processing, one of the core features of MS cognitive dysfunction. Some research has been conducted on its use in MS [45]. The MACFIMS, on the other hand, was developed specifically for people with MS and based on professional consensus. It focuses specifically on the areas of cognition commonly affected in MS. As the MACFIMS is a longer battery, the BICAMS is recommended for clinicians who may not have the time to administer the entire MACFIMS with patients [9]. A recent survey of 56 HCPs from the Consortium of MS Centres (CMSC) indicated that the BICAMS was only used by 14.3% of those surveyed [43]. This is surprisingly low considering these HCPS are members of the CMSC and working with a large MS caseload. Nevertheless, the variety of assessments used by clinicians in the current study highlights the need for clearer assessment pathways and guidelines in cognitive clinical care, as well as training for HCPs.

The evidence-base for cognitive rehabilitation in MS has grown substantially in the past 10 years, for example, a Cochrane review of memory rehabilitation for people with multiple sclerosis [24] found encouraging results in various areas of cognition and in improving outcomes for patients with MS. Despite the promising evidence emerging from the research, over 75% of the respondents in the current survey stated that their decision making for cognitive interventions was guided by clinical judgement, rather than evidence-based recommendations. Although clinical judgement is an important corner stone of evidence-based practice, it should be used in conjunction with the best available and up-to-date evidence and patient preferences [46, 47]. Feedback from people with MS who receive cognitive input from HCPs [48] indicates that the way in which information is provided and communicated can often be difficult for people who are already experiencing cognitive challenges. This is another area that needs to be considered by HCPs providing cognitive care to people with MS and is linked to the “teaching approaches” presented in Table 5.

Worryingly, regardless of the high numbers of people with MS who will experience cognitive difficulties, 36% of HCPs report not providing information or services in relation to cognition to people with MS. In addition, only 34% reported routine screening of cognition within their services and 39% reported not referring elsewhere when cognitive difficulties were suspected. Given the substantial impacts that cognitive difficulties can have on daily life [2, 5, 7], it is concerning that this area is being neglected in many services. The reasons for this were not clear from the survey, though some qualitative feedback suggests this may be as a result of not having referral options available, limited resources, long waiting lists, or the clinical expertise in MS and/or cognition within teams. Similar to this [48], in a survey of 27 HCPs at a recent education event, it was reported that HCPs did not believe that cognition as well by health care services or well-resourced in the UK. These results also reflect those reported by Klein et al. [15].

Despite the low level of attention to cognition in practice, clinicians rate its importance as very high with respect to both treatment and assessment. In contrast, confidence in treatment and assessment of cognition was much lower across the HCPs surveyed, suggesting that a lack of confidence may be impacting on the cognitive care provided across services. Globally, there is also an increased recognition for the need to assess and treat cognitive difficulties in MS [43, 48]. From the open-text responses, HCPs indicated a desire for further specialist training and support in this area. Post-registration training has been limited for some of the HCPs and both specific training and peer support were requested by participants. Langdon et al. [48] also reported that HCPs requested further education and information for HCPs and those living with MS in order to improve cognitive care for patients. In the current study, there was also a need for increased resources and regional service availability reported. The unmet needs of people with MS identified some years ago [49] appear to still be present.

The assessment and treatment of mood difficulties appears to be higher than that seen in cognition, with 47% of people reporting mood assessment and 70% referring onwards if an issue was identified in the assessment. This may be because referral pathways are better developed in this area and guidance for HCPs may be clearer. However, 49% of HCPs did not provide any information to people with MS regarding mood, mental health, or psychosocial difficulties. Also, 23 participant respondents identified that either did not assess for mood, were unsure of how to assess for mood, or did so via informal questioning. There was also a range of nonspecific measures identified by HCPs in the assessment of mood. The HADS, PHQ, GAD7, and the BDI-II have been established as valid measures of mood in MS. The BAI is considered less valid due to the reliance on physical symptoms of anxiety [31, 33] but was reported to be used by some HCPs in the current study. Given the prevalence of mood and mental health difficulties in the MS population and its complex interplay with cognition and emerging evidence of positive outcomes for psychological therapies in managing mood in MS, further training and education in MS-specific assessment and management of mood is needed.

### 4.1. Limitations

The timing of data collection was impacted by a number of major events–the significant cyberattack on the HSE and the COVID-19 pandemic. The cyberattack meant that potential participants may have not received information on the survey because they did not have Internet access at work or access to their work e-mail. Though postal copies of the questionnaire were distributed, it is worth noting that many HCPs were working remotely (because of cyberattack and COVID-19) and were away from their physical offices.

The results may also be limited because of the imbalance of responses across healthcare professionals. A number of healthcare professionals who work closely with people with MS (e.g., nurses) were underrepresented in the survey. These HCPs may have felt that the research study was not targeted towards them, perhaps because they do not work exclusively with people with MS (or in the area of cognitive care in MS). Again, this may have been because of access to Internet and additional demands on staff because of the COVID-19 pandemic. Nevertheless, although the sample was relatively small, the numbers surveyed are proportional to the UK sample [15] and much higher than those reported in similar research [43].

Finally, this questionnaire did not specifically ask HCPs whether they used pharmacological interventions for cognitive difficulty in MS. Gromisch et al. [43] report that 39% of the HCPs they surveyed would consider using medications to address cognition and so it would have been interesting to see a comparison in this Irish sample.

## 5. Conclusion

It is clear from the research findings that high importance is placed on cognitive and psychosocial care in people with MS by healthcare professionals in Ireland. Despite this, our findings highlight that there are significant gaps in how HCPs address the challenges faced by people with MS in Ireland in relation to both cognition and mood. Decisions around clinical management appear to be mainly guided by clinical judgement, despite growing evidence-base in these areas. There appears to be little consistency in treatment and assessment across services. The difficulties in the translation of best evidence to clinical care appear to be linked to staffing and service limitations, lack of access to specialist training, and insufficient resources. Healthcare professionals appear to be motivated to improve service provision for people with MS but supports, training, funding, and clear clinical pathways need to be in place. The current study is an important progression in developing a means of standardizing care and pathways, at a European level, for people with MS-associated cognitive and mood difficulties.

## Figures and Tables

**Table 1:** The number of years healthcare professionals reported they had been working with people with MS.

Years working with people with MS	*n*
30+ years	2
25–29	2
20–24	10
15–19	18
10–14	12
5–9	13
1–4	23
Under one year	3
Very few MS clients within a period of practice	3

**Table 2:** Reported MS-relevant post-registration training completed by healthcare professionals^*∗*^.

Training listed	*n*
Administering and interpreting cognitive tests	34
Cognitive rehabilitation theory	24
Delivering cognitive rehabilitation techniques	24
Administering and interpreting neuropsychological assessment	18
Identifying cognitive impairment in MS	17
Assessing for mood or psychosocial difficulties	16
Interventions for mood or psychological difficulties	11
None	7

**Table 3:** Issues HCPs reported offering support and services for people with MS with.

Reported issues and services offered	Number	% of participants reporting service
Mobility	85	89
Falls	82	86
Fatigue	70	74
Cognition	62	65
Pain	59	62
Muscle tone	56	59
Continence	55	58
Mood	53	56
Health promotion	52	55
Communication problems	48	51
Respiratory problems	39	41
Visual problems	36	38
Sexual dysfunction	23	24
Other	14	15

**Table 4:** Healthcare professionals conducting cognitive assessment^*∗*^.

Healthcare professional	Number
Occupational therapist	56
Psychologist (neuro, clinical, trainee, and assistant)	40
Nurse (MS specialist and general)	22
Doctor (neurologist, GP, or trainee)	12
Speech and language therapist	4
Physiotherapist	3

**Table 5:** Strategies and approaches used by HCPs in cognitive intervention with people with MS.

Strategies internal (*n*)		Strategies external (*n*)		Teaching approaches (*n*)	
Repetition and rehearsal	36	Diary	79	Verbal encouragement	78
Associations	33	Calendar	75	Written instructions	35
Chunking	28	Mobile phone/tablet	66	Provide homework	30
Attention training	28	Notebook	61	Use functional activities to practice strategy	28
Visual imagery	27	Alarm	54	Practice strategy in rehabilitation sessions	28
Categorization	18	Notice board	50	Fatigue management approaches	4
“What, where, who, when, how?” technique	18	Post-it notes	41	Follow manual	3
Mnemonics	15	Dictaphone	10	Develop a game	2
Story method	13	Sleep hygiene	2	None provided	2
Mental notes	12	Removing distractions from environment	2	Promoting healthy diet and exercise	2
Awareness of big and small detail technique	10	Mobile applications	2	Involve family	1
Rhymes or songs	8	Structure and routine	2	Help patient identify own strategies	1

**Table 6:** HCPs reported in a survey who assess mood in people with MS.

Healthcare professional	Number
Nurse (MS specialist and general)	23
Psychologist (neuro, clinical, trainee, and assistant)	19
Doctor (neurologist, GP, or trainee)	14
Occupational therapist	11
Healthcare professional that has first contact with the patient	5
Physiotherapist	4
Unsure	4

## Data Availability

The data are available on request from the corresponding author. Part of this research was presented at the 2021 ECTRIMS congress [50].

## Additional Points

What is already known about this topic? Little is known about what constitutes ‘usual care' of cognitive impairment and the psychosocial needs of people with multiple sclerosis (MS) in Ireland. Though recent research found great variety in the assessment of cognitive symptoms in people with MS across the UK, with 50 different assessments reported as being used [15], a greater understanding of the assessment and treatment of cognitive impairments is requisite in an Irish context. What does this article add? The current study replicated a research study by Klein and colleagues [15] to better understand the assessment and treatment of cognitive impairments, with results suggesting that there is little consistency in treatment and assessment across services for people with MS in Ireland; with HCPs further indicating a desire for further specialist training and support in this area, as well as increased service availability. The current study is an important progression in developing a means of standardizing care and pathways, at a European level, for people with MS-associated cognitive difficulties.

## Supplementary Materials

Supplementary Materials Appendix 1 contains additional survey responses. It contains the cognitive assessments that are listed fewer than three times and the psychosocial assessments listed once.
