# Supplementary material for: Assessment and Management of Cognitive and Psychosocial Difficulties for People with Multiple Sclerosis in Ireland: A National Survey of Clinical Practice
Source: Int J Clin Pract. 2022 Oct 21;2022:3232076. doi: 10.1155/2022/3232076 (PMC9616662; doi:10.1155/2022/3232076)
Supplement: Supplementary Materials — Appendix 1 contains additional survey responses. It contains the cognitive assessments that are listed fewer than three times and the psychosocial assessments listed once. [file 3232076.f1.docx]

**Appendix 1 Additional survey responses**

***Other MDT members named***

Other MDT professions listed were: public health nurse (n=1), audiologist (n=1), counsellor (n=1), clinical nurse specialist continence advisor (n=1), pharmacist (n=1), clinical engineer (n=1), GP (n=1), occupational therapy assistant (n=1), physiotherapy assistant (n=1), and speech and language therapy assistant (n=1).

**Other services provided to people with MS (linked to Table 3)**

Other areas listed were: assistive technology and specialised seating (n=1); audio-vestibular diagnostics (n=1), unsure (n=1), work (n=1), Functional Electrical Stimulation (n=1), casework (n=1), balance (n=1), respite services (n=1).

***Cognitive assessments***

Those listed by less than three HCPs were:

Test your memory, Cognitive Assessment of Minnesota, Boston Naming Test, Multiple Errands Test, Test of Everyday Attention, Paced Auditory Serial Addition Test, Sydney Language Battery, The Motor-Free Visual Perception Test, Automated Neuropsychological Assessment Metrics, Speed and Capacity of Language Processing Test, The Visual Object and Space Perception Battery, Pyramids and Palm trees, Rey Complex Figure Test, Western Aphasia Battery, Six-item cognitive impairment test, 7/24 Spatial recall test, The Brixton Spatial Anticipation Test, Rey Auditory Verbal Learning Test, The Loewenstein Occupational Therapy Cognitive Assessment, and The Adaptive Behavior Assessment System.

***Psychosocial assessment***

Other assessments, mentioned once, were:

Visual Analog Mood Scales, Personality Assessment Inventory, Mental State Examination, 4AT- Rapid Clinical Test for Delirium, Edinburgh Postnatal Depression Scale, COPE Inventory, Mental Health Inventory, Modified Fatigue Impact Scale, K methods, Core – 10 Depression scale, Geriatric depression scale, SF-36, Generalised Anxiety Disorder Assessment 7.
